# Case report: Bullous pemphigoid combined with Sjögren’s syndrome complicated by central nervous system infection

**DOI:** 10.3389/fimmu.2024.1419054

**Published:** 2024-09-10

**Authors:** Xing-Yue Chen, Jun Chen, Kun-Lan Long, Peng Ding, Rong Li, Li-Jia Zhi

**Affiliations:** ^1^ Department of Critical Care Medicine, Hospital of Chengdu University of Traditional Chinese Medicine, Chengdu, China; ^2^ Department of Critical Care Medicine, Deyang Hospital Affiliated Hospital of Chengdu University of Traditional Chinese Medicine, Chengdu, China; ^3^ School of Clinical Medicine, Chengdu University of Traditional Chinese Medicine, Chengdu, China

**Keywords:** bullous pemphigoid, Sjogren’s syndrome, pathological mechanisms, autoimmune diseases, central nervous system infection

## Abstract

**Background:**

Bullous pemphigoid (BP) is the most common autoimmune blistering skin disease in humans, characterized by tense blisters, erosions, urticarial lesions, and itching on normal or erythematous skin. Many autoimmune diseases are considered comorbidities of BP, but clinical case reports of BP complicated by Sjögren’s syndrome are very scarce. Furthermore, cases of central nervous system infection secondary to both autoimmune diseases are even rarer.

**Case presentation:**

We report a 74-year-old woman diagnosed with bullous pemphigoid, who showed relief of active lesions after treatment with methylprednisolone and dupilumab injections. However, she was admitted for pulmonary infection during which she was diagnosed with Sjögren’s syndrome (SS). Subsequently, the patient developed altered consciousness, indicating a central nervous system infection. Adjustment of steroid dosage and aggressive antimicrobial therapy led to alleviation of symptoms.

**Conclusion:**

The coexistence of autoimmune subepidermal blistering diseases and SS is rare. The role of SS in the pathogenesis of skin lesions is unclear, and the relationship between these blistering diseases and SS remains elusive. Further research is needed to determine whether there are common pathological mechanisms between the two conditions.

## Introduction

Bullous pemphigoid (BP) is the most common autoimmune blistering disease, primarily affecting the elderly, with peak onset around 80 years of age, and no significant gender difference (1). Clinical manifestations mainly include erythematous urticarial plaques, blisters, and intense itching. It is induced by autoantibodies against two dermal-epidermal junction proteins, BP180 and BP230. Binding of autoantibodies leads to complement activation, inflammatory reactions, degranulation of mast cells, accumulation of neutrophils and eosinophils, and release of proteases cleaving BP180, resulting in blister formation (2). The pathogenesis of BP is largely unknown, but previous cases have been associated with specific “potential triggers” such as drugs (3, 4), physical factors, vaccines (5), infections, and transplants. The gold standard for BP diagnosis is confirmation of linear deposition of IgG and/or C3 along the dermal-epidermal junction by direct immunofluorescence (DIF). In recent years, numerous autoimmune diseases have been reported as comorbidities of BP, including neurological conditions (such as multiple sclerosis, dementia, Parkinson’s disease, epilepsy, stroke) and cardiovascular diseases (such as diabetes, hypertension, pneumonia, pulmonary embolism), with an increased incidence of comorbidities in BP patients (6–8). Although Sjögren’s syndrome (SS) is often associated with other autoimmune diseases, occasional reports exist of its coexistence with different autoimmune blistering skin disorders. However, the coexistence of SS and autoimmune subepidermal blistering diseases is rare, with only one case report by Yamamoto T et al. in 1998 documenting the co-occurrence of pemphigus foliaceus and Sjögren’s syndrome, further reducing the occurrence of rare central nervous system infections.

## Case description

**Figure 1:**
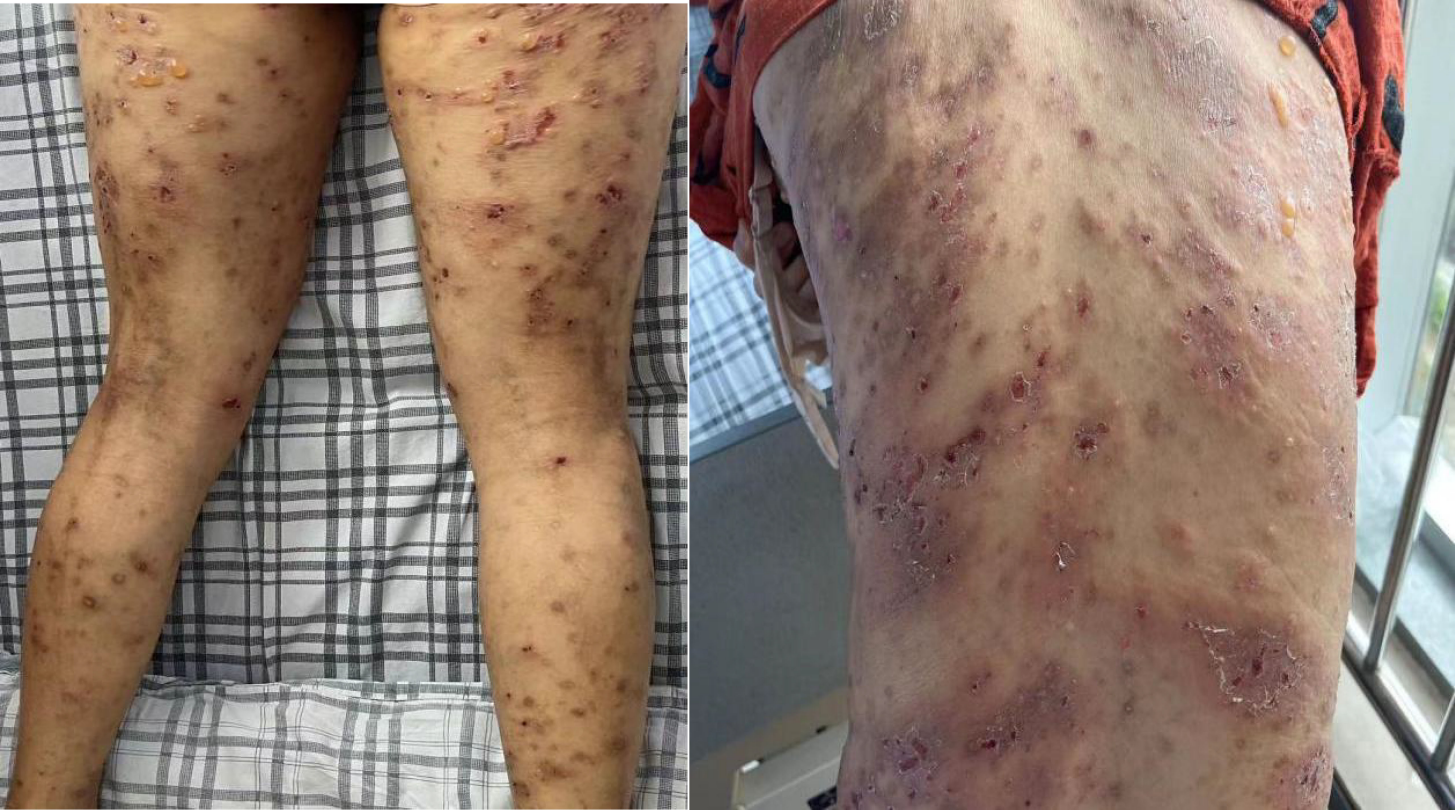
Erythematous papules, pustules, and vesicles all over the body.

**Figure 2:**
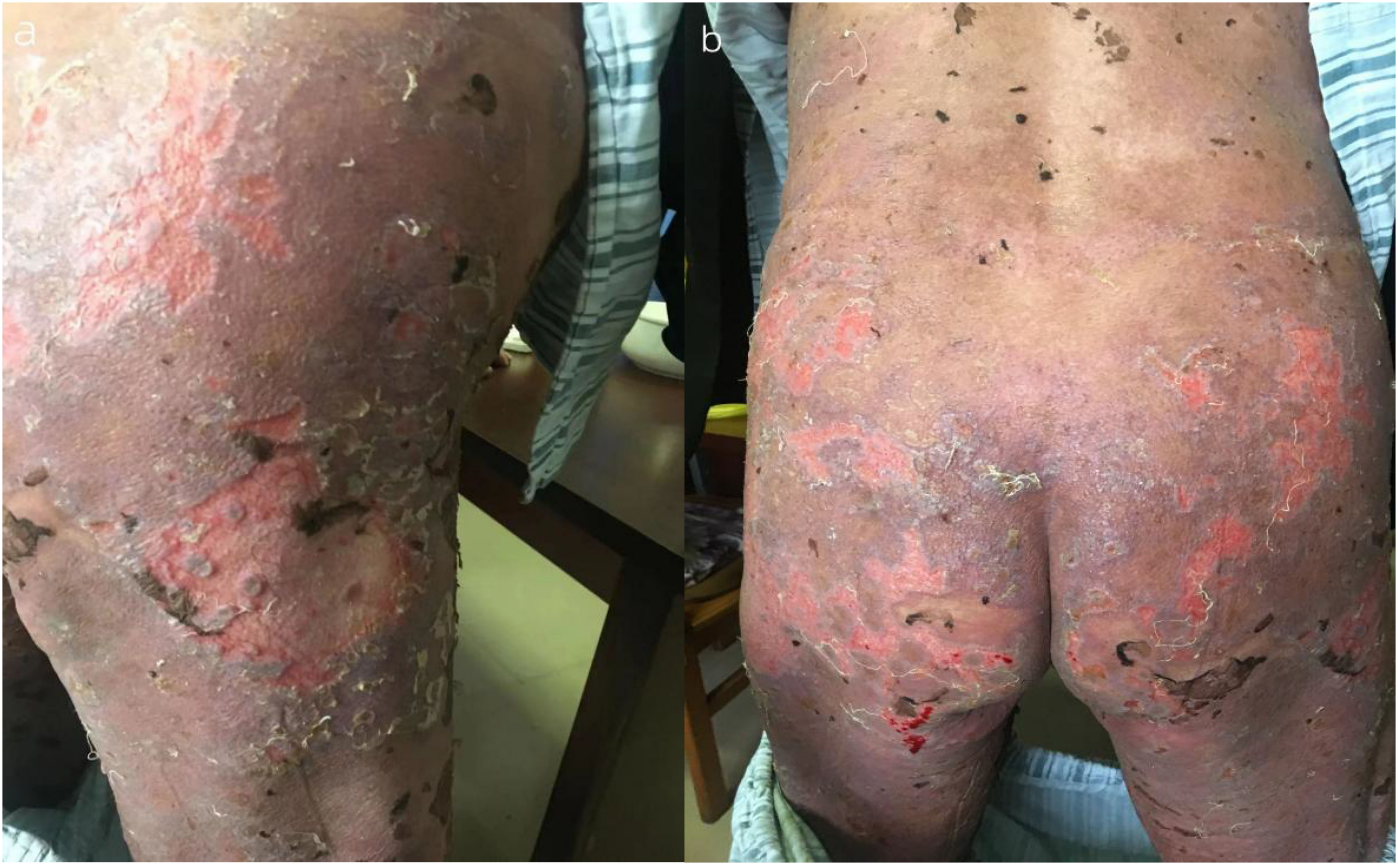
**(A, B)** The buttocks and lower limbs are crusted and scabbed.

**Figure 3:**
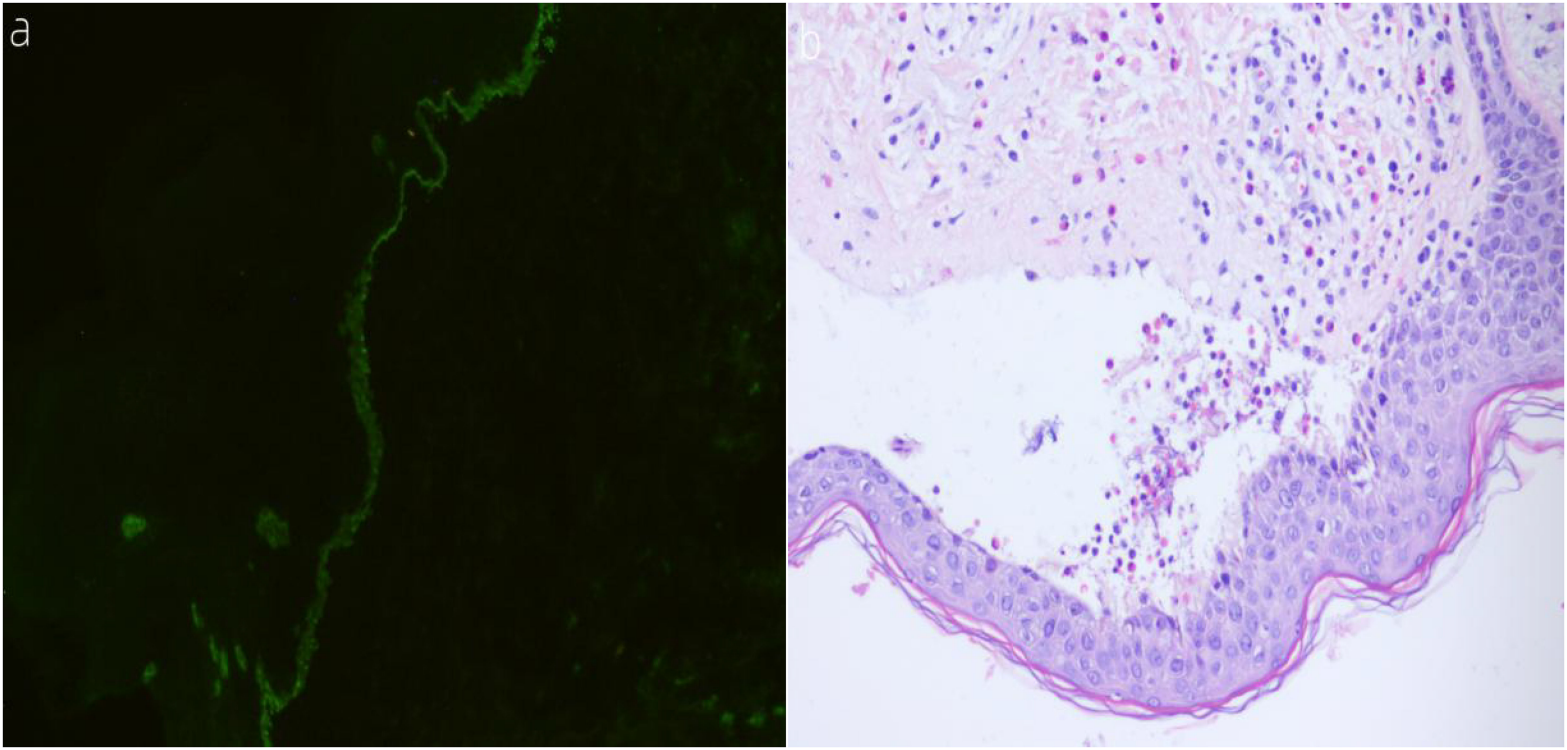
**(A, B) (A)** Direct immunofluorescence (DIF) shows linear deposition of IgG along the dermoepidermal junction at the periphery of the lesions. **(B)** Histological features on H&E staining reveal abundant infiltration of eosinophils.

A 74-year-old woman presented with widespread erythematous patches and vesicles on the limbs, which had appeared successively over a period of 2 weeks, and had previously been diagnosed as “eczema” at another hospital. Treatment with acyclovir cream was ineffective. Two days before admission, the patient’s condition worsened, prompting a visit to our dermatology department. On admission, the patient exhibited erythematous papules, pustules (**Figure 1**), particularly severe on the buttocks and both lower limbs, which were ulcerated with yellowish crusts. The total area of skin lesions was approximately 1000 cm2, with an exudation area of approximately 200 cm2 (**Figures 2A, B**). The patient had no past medical history, no history of allergies, no history of medication use, and no history of exposure to harmful substances. Laboratory investigations did not reveal any significant abnormalities. For a definitive diagnosis, direct immunofluorescence (DIF) was performed on the skin lesions, showing linear deposits of IgG along the dermoepidermal junction, histological features consistent with HE staining, and significant infiltration of eosinophils (**Figures 3A, B**). Serum anti-bp180 antibody testing was positive, confirming a diagnosis of BP. The patient was treated with methylprednisolone 40 mg orally per day, which was adjusted to methylprednisolone tablets orally after one week, with a dose of 20 mg in the morning and 16 mg in the evening. The patient’s overall skin condition significantly improved, and she was discharged after two weeks. The dosage of methylprednisolone tablets was gradually reduced after discharge.

Two months later, the patient developed a small number of new skin lesions throughout her body, prompting readmission to our dermatology department. The methylprednisolone dosage was adjusted to 16 mg in the morning and 12 mg in the evening, and dupilumab injection was added. Dupilumab was administered subcutaneously, with an initial dose of 600 mg (300 mg injected twice), followed by 300 mg every two weeks. Following treatment, the skin lesions partially crusted over. The patient was discharged after a week of hospitalization and continued to take methylprednisolone tablets orally at the original dosage regimen, with regular subcutaneous injections of dupilumab.

**Figure 4:**
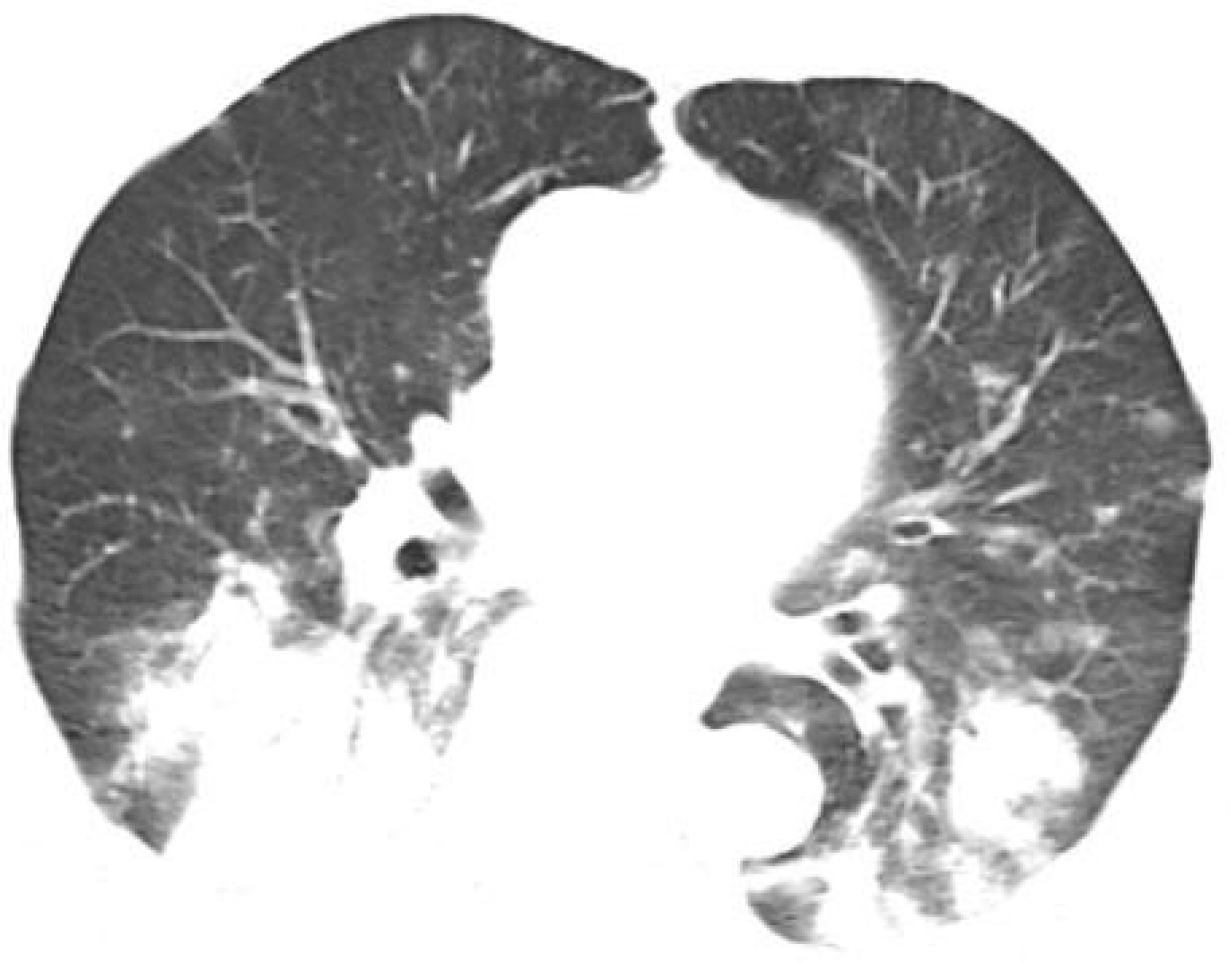
Chest CT scan shows increased lung markings with scattered patchy opacities.

**Figure 5:**
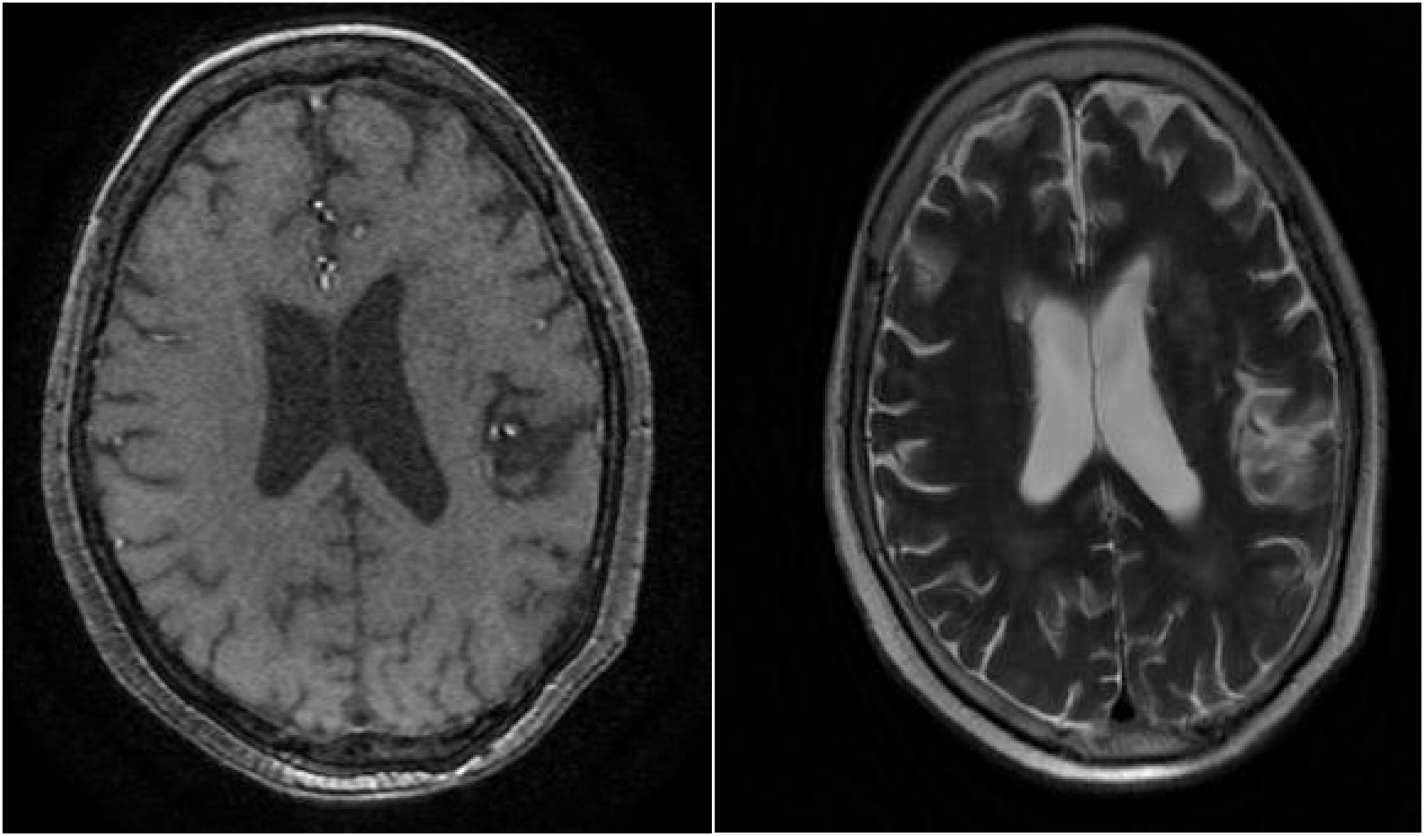
Cranial MRI reveals several punctate low-density lesions in bilateral basal ganglia regions, with slightly deepened and widened cerebral sulci and fissures, centrally located, with no other significant abnormalities observed.

However, one month later, the patient developed symptoms of fatigue and dyspnea, leading to admission to our cardiology department. The patient’s symptoms worsened gradually, eventually resulting in respiratory failure, necessitating transfer to the intensive care unit. Chest CT revealed patchy changes (**Figure 4**), while head CT and MRI showed no significant abnormalities (**Figures 5**). High-throughput pathogenic microorganism sequencing in peripheral blood (Next Generation Sequencing, NGS) suggested the presence of human herpesvirus 3 (VZV), human herpesvirus 5 (CMV), human herpesvirus 1 (HSV1), and human herpesvirus 4 (EBV), with viral sequence counts exceeding 100,000 (**Table 1**). Therefore, the patient was diagnosed with viral septicemia and treated with acyclovir for antiviral therapy. Since no new skin rash was observed in this instance and considering the severely compromised immune status due to severe infection, the methylprednisolone dosage was reduced to 4 mg, and intravenous immunoglobulin was added to boost immunity after consultation with the dermatology department. However, the patient’s autoimmune antibody spectrum indicated positive anti-recombinant RO-52 antibodies, anti-nuclear antibodies, and anti-SSA antibodies (**Table 2**). Further inquiry into the medical history revealed noticeable symptoms of dry eyes and dry mouth reported by the patient’s family over the past two months. To confirm the diagnosis, a Schirmer test showed a positive result. According to diagnostic criteria established by Vitali C (9), the patient was diagnosed with Secondary Sjögren’s Syndrome. Meanwhile, during the course of the illness, the patient gradually developed a decreased level of consciousness. Initial head MRI showed no significant changes. Subsequent lumbar puncture revealed Enterococcus and human herpesvirus 7 in the cerebrospinal fluid. Considering that the patient suffered from severe immunosuppression, with reactivation of several herpesviruses in plasma, before having a microbial CNS infection and succumbing to lung infection. We prescribed broad-spectrum antimicrobial therapy with meropenem, linezolid, and acyclovir. Human immunoglobulin was administered intravenously at a dose of 20 g once daily for 5 consecutive days. Additionally, thymosin alpha-1 was used to boost T cells and the overall immune system, with a daily subcutaneous injection of 1.6 mg for 1 week. During this period, the patient’s immune function improved compared to before (**Table 3**). After active treatment, the patient’s condition improved, and she was transferred out of the ICU, but her consciousness level remained incomplete, characterized by the ability to open eyes spontaneously but inability to follow commands. Upon follow-up two months later, the patient succumbed to complications from severe secondary pulmonary infection.

**Table 1:** High-throughput pathogen microbial sequencing of peripheral blood.

Type	Species Name	Sequence Count	Estimated Pathogen Concentration (copies/ml)
virus	VZV	226074	10^5^
virus	CMV	209119	10^5^
virus	HSV1	143155	10^5^
virus	EBV	109419	10^5^

VZV, human herpesvirus 3; CMV, human herpesvirus 5; HSV1, human herpesvirus 1; EBV, human herpesvirus 4.

**Table 2:** Autoimmune antibody profile.

Autoantibody	Result
Anti-pm-scl antibody	Negative
Anti-mitochondrial M2 antibody	Negative
Anti-ribosome P protein antibody	Negative
Anti-histone antibodies	Negative
Anti-nucleosome antibody	Negative
Anti-cyclin antibody	Negative
Anti-centromere B antibody	Negative
Anti-jo-1 antibody	Negative
Anti-scl-70 antibody	Negative
Anti-SSB antibody	Negative
Anti-recombinant RO-52 antibody	positive(+)
Anti-SSA antibody	positive(+)
Anti-Sm antibody	Negative
Anti-U1RNP antibody	Negative
Anti-double-stranded DNA antibody	Negative
Anti-cyclic citrulline peptide antibody	Negative
Rheumatoid factor	Negative
Antinuclear antibody	positive(+)

**Table 3:** Changes in immune system markers.

Marker	D1	D2	D3	D4	D5	Reference range
IgG(g/L)	4.70	8.43	12.2	11.60	15.43	7.51-15.60
IgA(g/L)	1.07	1.25	1.16	1.69	1.24	0.82-4.53
IgM(g/L)	0.66	0.72	0.63	0.81	0.76	0.46-3.04
Total T lymphocyte count (cells/μL)	104	137	311	472	432	683
Total T lymphocyte percentage (%)	54.25	60.45	71.50	68.43	64.21	76.31
B lymphocyte count (cells/μL)	65	79	303	384	471	85-616
NK Cell Count(cells/μL)	19	44	129	261	289	84-724

IgG, Immunoglobulin G; IgA, Immunoglobulin A; IgM, Immunoglobulin M; NK, Natural Killer Cell.

## Discussion

BP is an autoimmune skin disease that predominantly affects the elderly, with few clearly defined etiologies, and it can be associated with various autoimmune disorders (10, 11). From an immunological and clinical perspective, this case can be regarded as a rare skin disease mediated by the correlation between autoantibodies. The patient exhibited only IgG antibodies against BP180 in active BP serum. After initial treatment with steroids, the systemic skin lesions improved; however, two months later, new lesions appeared. Considering the refractory and difficult-to-treat nature of BP, the Th2 pathway is now recognized as a major triggering factor for BP antibody production (12). In recent years, researchers have proposed anti-IL-4Rα as a potential therapeutic approach for these diseases (13, 14). Dupilumab is a fully humanized IgG4 monoclonal antibody that specifically binds to the IL-4Rα subunit, thereby inhibiting the signaling of IL-4 and IL-13, blocking the type II inflammatory response mediated by IL-4 and IL-13, and achieving good results in the treatment of specific skin inflammations (15). According to reports, dupilumab can significantly reduce the serum levels of CCL17, which is a key regulatory factor in the Th2 immune response (16). Dupilumab treatment not only alleviates itching and improves skin lesions but also normalizes anti-BP180 antibodies (17). In the only multicenter case series reported, 92.3% of patients achieved disease remission or satisfactory responses, with 53.8% of patients achieving complete clearance of BP (18). In this case, the patient showed a significant decrease in BP180 IgG antibodies after treatment with steroids and dupilumab, maintaining levels below the critical threshold during remission, demonstrating the effectiveness of BP treatment. However, the long-term efficacy of dupilumab for BP patients remains to be further clinically verified, and more research is needed to determine the optimal dosage and treatment intervals to maximize benefits. This patient developed SS after the improvement of BP, and the relationship between SS and BP is not yet clear. Therefore, in this case, Sjögren’s syndrome can occur before, simultaneously with, or after the onset of BP. For the coexistence of these two autoimmune diseases, the most acceptable hypothesis is epitope spreading, whereby tissue damage exposes target antigens to autoantibodies during inflammation, potentially leading to secondary autoimmune diseases in certain cases (19). We speculate that the combination of BP and SS may be due to epitope spreading phenomena. Regarding epitope spreading, we discuss the possibility of homogeneity or proximity of target molecules or epitopes of autoantibodies. Epitope spreading refers to the variability of autoimmune reaction targets, extending to other epitopes of the same protein or other proteins within the same tissue. It can also result from tissue damage caused by primary inflammatory processes, leading to the release and exposure of previously “isolated” antigens, triggering secondary autoimmune reactions against newly released antigens (20). We speculate that the immune response of BP may influence the progression of SS to some extent. Regarding the correlation between the two, we propose the following hypotheses. On one hand, similar to other autoimmune diseases, polymorphisms in certain genes may play a role in the pathogenesis of BP. Considering that genetic susceptibility is hereditary, even in familiar genetic backgrounds, susceptibility to one autoimmune disease may predict susceptibility to another (19). While acknowledging the impact of genetics on autoimmune diseases, we must also consider other factors that may lead to autoimmunity. Self-activated Th1, Th2, and B cells can target different structural domains of BP180, leading to the production of autoantibodies against BP180 through epitope spreading and Ig class switching. Such autoantibodies can persist in the serum long before clinical symptoms appear. The attacked BP180 can become a source of new antigens, further amplifying the production of autoantibodies and accelerating the disease (21). The MHC gene is located in a highly polymorphic region on the short arm of chromosome 6 (6p21) and is an important component of the immune response. Three classes of molecules within the MHC are class I (HLA-A, -B, and -C), class II (HLA-DR, -DP, and -DQ), and class III (complement and cytokine genes) (22). It has been reported that in population studies, HLA class II alleles are associated with BP in several ethnic groups, including British, German, Japanese, Chinese, and Iranian populations (23–26), with significant clinical variation. These HLA risk alleles are also associated with SS patients, who often have higher serological activity and spreading of antibodies to Ro/La autoantigens, rather than maintaining a single positivity for anti-ro52 or anti-ro60 (27). These high-risk HLA alleles may be associated with the ability of T cells to recognize and participate in Ro/La autoantigen. Therefore, we speculate that HLA genes may be a common genetic background between these diseases, leading to frequent autoimmune reactions of SS patients to BP180. On the other hand, studies have shown that Th17 cells are involved in exacerbating the inflammatory response of BP, promoting the activation of autoreactive T cells and the production of autoantibodies, together with dysregulated regulatory T cells (Tregs). Immunohistochemical studies have shown an increase in Th17 and Treg cells in BP-affected skin (28–32). In SS mouse models, enhanced expression of IL-17/IL-23 suggests Th17 involvement in salivary gland lymphocyte infiltration and lesion formation (33, 34). Elevated levels of IL-17 in BP blister fluid suggest its crucial role in eosinophil infiltration and subsequent BMZ damage (28, 35). Meanwhile, SS patients show increased levels of IL-22, IL-23, IL-17 protein, and mRNA in peripheral blood. Between BP and SS, impaired function and stability of Treg cells, along with abnormal induction and proliferation of Th17 cells, may drive the activation of other immune cells, potentially facilitating the mutual transformation and spread of acute autoimmune diseases. Chiu (36) found through a large-scale study of the Taiwanese population that BP patients are prone not only to psoriasis and SLE but also to SS and alopecia areata, among other autoimmune diseases, with BP-SS comorbidity being significant only in female patients, for reasons that remain unclear. The coexistence of multiple autoimmune diseases is not incidental and may involve underlying pathogenic mechanisms, necessitating further research to explore whether there is a potential correlation between their occurrence and development. The mutual amplification of inflammatory processes resulting from multiple factors leading to immune dysfunction may contribute to disease variability. In this case, the patient developed a central nervous system infection, which may be related to glucocorticoid use, immune system abnormalities, and immunosuppressive therapy, leading to such opportunistic infections, and was also associated with the reactivation of multiple herpesviruses in the blood. However, whether dupilumab exacerbated this process has not been reported in the literature. When the patient was transferred out of the Intensive Care Unit (ICU), partial recovery of consciousness was observed, with the ability to open eyes spontaneously but an inability to follow commands. This may be related to CNS infection or neurological diseases secondary to BP. Previous studies have suggested a close association between BP and neurodegenerative diseases, with BP patients being more prone to cognitive impairment (37–39). BP180 and BP230 are expressed in human brain tissue, with BP180 mainly distributed in neuronal cell bodies and proximal axons. The age-related weakening of the blood-brain barrier and the loss of brain immune tolerance expose BP180 and other neural tissue autoantigens, which may be the mechanism for the development of CNS neurodegenerative diseases after BP (40). However, a consensus has not yet been reached on this matter. Due to the relatively few cases of BP complicated by autoimmune diseases and the lack of related research, the pathogenesis remains unclear. To our knowledge, there are no reported cases of BP complicated by SS. Therefore, the possible mechanisms discussed in this paper are hypotheses or speculations and have not been confirmed, requiring further in-depth research. This case provides insight that, in clinical practice, early screening for relevant immune complications based on the medical history, clinical manifestations, and past history of BP patients is crucial. The possibility of purely coincidental findings in elderly patients is not remote. SS may develop with minimal symptoms over many years. Timely detection and treatment are essential to prevent missed diagnoses and treatment delays, thereby improving patient prognosis. In addition, treatment regimens should be adjusted according to the different complications of BP to avoid mutual interference. However, longer-term follow-up and more cases are needed for validation.

## Data Availability

The raw data supporting the conclusions of this article will be made available by the authors, without undue reservation.
